# Effectiveness of Electroconvulsive Therapy for Depression and Cotard's Syndrome in a Patient with Frontotemporal Lobe Dementia

**DOI:** 10.1155/2012/627460

**Published:** 2012-10-17

**Authors:** Toshiyuki Kobayashi, Koju Inoue, Katsutoshi Shioda, Satoshi Kato

**Affiliations:** Department of Psychiatry, Jichi Medical University, 3311-1 Yakushiji, Tochigi, Shimotsuke 329-0498, Japan

## Abstract

In the field of psychogeriatrics, the differential diagnosis of depression and dementia, as well as the treatment of depression and comorbid dementia, is an important issue. In this paper, the authors present the case of a 72-year-old woman with Cotard's syndrome and frontotemporal dementia (FTD) who was admitted to a psychiatric hospital with delusions of negation accompanied by depressive symptoms. Pharmacotherapy over a 2-year hospitalization was unsuccessful, and she was subsequently transferred to our university hospital. A total of 18 sessions of electroconvulsive therapy released her from psychomotor inhibition, appetite loss, and Cotard's delusions. The indication for electroconvulsive therapy in patients with dementia is discussed.

## 1. Introduction

Depression and dementia are among the most common disorders seen in mental health clinical practice for elderly people with mental disorders. The conundrum is that both disorders sometimes coexist, can sometimes succeed each other, and hence can often confuse clinicians [1–3]. Even though we do not have sufficient clinical tools to cure dementia, depression is thought to be treatable. We can try to relieve the distress of patients with this comorbidity by at least treating the depression. However, elderly patients with depression often have somatic and/or psychiatric disease, an atypical clinical picture, and sensitivity to environmental or psychological factors [2] and are often resistant to pharmacotherapy or show adverse effects with antidepressants. In such cases, electroconvulsive therapy (ECT) can become an important option [1].

 In this paper, we present a case of a senile patient with marked inhibition, appetite loss, and delusions similar to Cotard's syndrome, who was almost bedridden for 2 years. She was diagnosed with frontotemporal dementia (FTD) with depression, but ECT released her from the depressive symptoms including inhibition that had led to her being bedridden.

## 2. Case Representation

A female, ex-blue-collar worker, and housewife gradually lost her willingness to work sometime in the spring at the age of 72 years. Although she had been a hard worker busy caring for others, she started washing only her own and not her family's dishes. She also started showing incomprehensible rigid behavior: when her husband was admitted to hospital, she doggedly kept to her daily routine of walking. She complained of loss of appetite and insomnia and was unwilling to go out and socialize. Her family took her to a neurologist, but neurological examination and brain computed tomography (CT) revealed no abnormalities.

 In the winter, she began to express suicidal ideation and her family took her to a psychiatric clinic. She was diagnosed with depression. However, she often refused to take her medication, withdrew into her shell, and stayed in bed all day long. 

 In January of the following year, she was admitted to a psychiatric hospital with depression. She had a glazed expression and answered all questions with “No good” or “I cannot.” She showed marked psychomotor inhibition. Her nutrient intake was insufficient and she presented with urinary incontinence unless taken to the toilet by staff. She showed delusions of negation with occasional megalomaniac features, for example, “My one lung does not exist,” “My stomach and intestines do not exist,” “I feel that I repeatedly died,” “I caused a scandal. I did wrong. I will be forever beyond forgiveness,” and “I destroyed Tokyo Tower.” During her stay of 2 years and 3 months at the psychiatric hospital, various medical regimens were tried but were unsuccessful: tricyclic antidepressants could not be increased because of side effects including delirium, severe constipation, and extrapyramidal symptoms; selective serotonin reuptake inhibitors had insufficient effects at the clinical dosage; the addition of atypical antipsychotics caused drowsiness; the addition of pramipexole dihydrochloride also caused marked drowsiness; augmentation with valproic acid was ineffective.

 At the age of 74, the patient was bedridden and could not take sufficient food. The patient's home and the psychiatric hospital are located in a remote area, and transferring her to a different hospital was burdensome for the family. Her family eventually agreed to transfer her to our hospital in April when the patient was at the age of 75 in order to determine the indication for ECT. 

**Figure 1:**
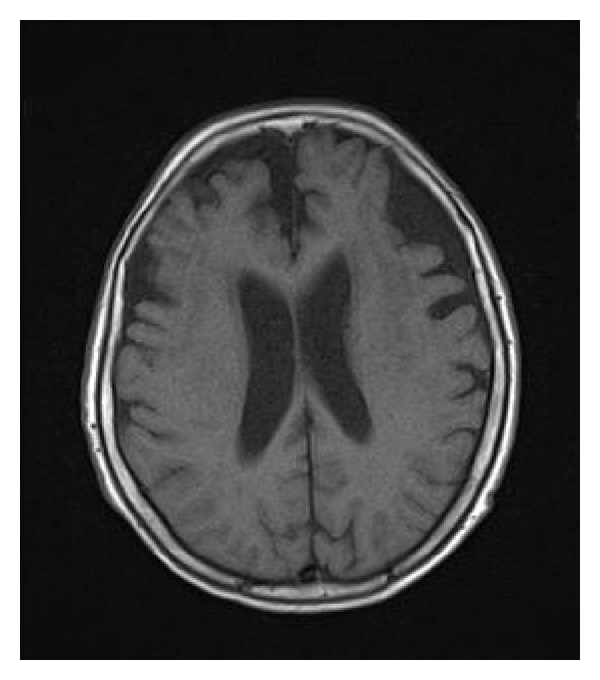
Magnetic resonance image on hospital day 10 shows moderate cortical atrophy especially in the frontal lobe.

**Figure 2:**
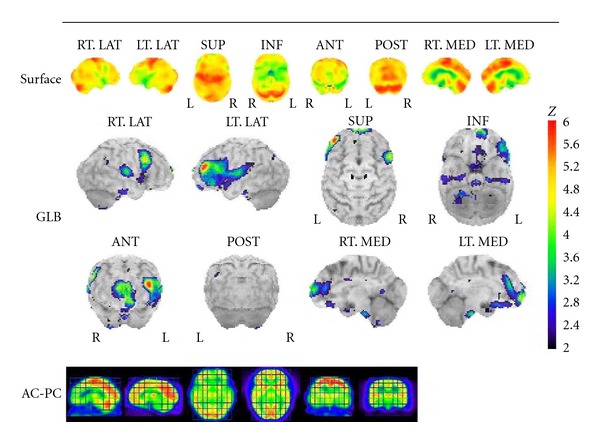
Three-dimensional stereotactic surface projections on brain single photon emission computed tomography show decreased blood flow in the frontal lobes, predominantly on the left side.

 On admission to our hospital, she exhibited several neurological signs suggesting frontal lobe disturbance, including the snout, sucking, grasping, and palmomental reflexes. Taking together her rigid and inappropriate behavior prior to her original hospitalization with these abnormal reflexes, we suspected she had FTD. She was found to have a Hasegawa Dementia Scale-Revised (HDS-R) [4] score of 7 (a score of 20 or less raises the suspicion of dementia), and a mini mental state examination (MMSE) score of 14. Brain CT and magnetic resonance imaging (MRI) revealed moderate cortical atrophy predominant in bilateral frontal lobes (Figure 1) without hippocampal atrophy. Single photon emission computed tomography (SPECT) of the brain showed decreased blood flow in the frontal lobe predominantly on the left side (she was right-handed, Figure 2). 

 Her inhibition was so severe that she lays motionless and made only single-word responses or no response. She ate half or less than half of her meals. Her spontaneous speech was typically, “No good,” “Can't eat,” and “Can't go to the toilet,” and sometimes she denied the existence of her heart, stomach, and intestines. We interpreted that “cannot” was a sign of depression—$Nicht\;\;k\ddot{o}nnen$ according to von Gebsattel [5]. She had been bedridden for more than 2 years, and pharmacotherapy demonstrated its limitations. Irrespective of the presence of FTD, it was likely that she had comorbid depression, and her condition was considered an indication for ECT.

 Between hospital days 11 and 47, the patient received a total of 12 ECT sessions. During the treatments, her mental condition fluctuated. Often after receiving treatment, on the same day her appetite was improved, and she acknowledged the existence of her heart or stomach; however, the following day she became anorexic and again denied the existence of her heart or stomach. Maintenance ECT sessions (around once a week) were continued until hospital day 77, because the effect of ECT lasted only about one week. Over time, her appetite loss, delusions of negation, slowness in response, and physical sluggishness were improved and then again deteriorated, although baseline mental and physical conditions were gradually improving. The content of her stereotyped speech changed from negation to “Hungry!” She became less anorexic, more active, and less negative, despite her cognitive function being impaired: HDS-R score was 6 and MMSE score was 13. She was transferred to the previous hospital on hospital day 82.

 In the hospital, she was still inactive but no longer bedridden. Over the course of the next year, however, she continued to show stereotyped speech and expressed suicidal ideation, but could use more expressive words. Symptoms suggesting dementia did not progress further during this period.

## 3. Discussion

To our knowledge, this is the first report on Cotard's syndrome associated with FTD. Only Fàzzari et al. have reported a similar positive outcome of ECT in a 69-year-old man with Cotard's syndrome and frontotemporal atrophy, not FTD [6]. Patients with FTD, extensively involving the brain, exhibit a broad range of psychopathology including mood disorders. Blass and Rabins divided depressive syndrome in FTD into 3 syndromes: major depression according to DSM-IV, a syndrome of mood lability with prominent responsiveness to the environment, and a syndrome of profound apathy [7]. This classification is clear-cut, but clinicians often have difficulty distinguishing depressive inhibition from apathy. 

 The diagnosis of FTD may be controversial in this patient. If the patient had FTD with comorbid depression, as we suppose, it is difficult to apply the clinical diagnostic criteria [8] to the patient. Her condition clearly met the core criteria for FTD [8]: insidious onset and gradual progression, early decline in social interpersonal conduct, early impairment in regulation of personal conduct, early emotional blunting, and early loss of insight. However, these can be explained by depression with severe inhibition. There were also some supportive diagnostic features [8]: decline in personal hygiene and grooming, mental rigidity and inflexibility, aspontaneity and economy of speech, stereotype of speech, mutism, and primitive reflexes. In addition, although the findings on brain CT, MRI, and SPECT are not typical findings of FTD, they do suggest frontal hypofunction which supports our diagnosis. It cannot be denied that she had Alzheimer's disease with atypical presentation; however, there were no findings strongly suggesting Alzheimer's disease. Dementia with Lewy bodies can be ruled out because there were few symptoms meeting the criteria [9]. The most prominent clinical feature was stereotyped speech, in which the patient declared her desire to eat is a reflection of stereotyped behavior and appetite increase that are suggestive of FTD [10, 11], which remained after ECT. Based on this clinical picture, FTD seems the most likely diagnosis.

 Our patient's early symptoms can be interpreted as apathy in the early phase of dementia concomitant with loss of energy due to depression. The relationship between depression and dementia in our patient is unclear. Did she first develop a depressive episode and then developed dementia? Or was dementia complicated by depression? Her clinical course suggests that she had depression with severe inhibition and Cotard's delusions. Her previous doctor made an effort to treat her depression. We initially suspected that her depression was refractory or that it was not depression but apathy due to FTD. She did not mention how she felt and mostly used stereotyped phrases that were not expressive, and she did not seem depressive. The fact that she became sloppy and gave priority to her daily routine over her husband's hospitalization in the early course strongly suggests personality change in FTD. However, her symptoms were not fully explainable by FTD because we could hear her depressive mood in her stereotyped words. Kobayashi and Kato conceptualized such an indistinguishable state as depression-dementia medius, a spectrum extending from depression without dementia symptoms to dementia without depression [3].

 One of the reasons why we thought she had comorbid depression was that she exhibited Cotard's delusions. Cotard's syndrome [12] was firstly described as a case of a lypemania (grossly equal to melancholy) with delusions of negation [13] and has been considered mainly a particular type of depression. However, Berrios and Luque [14] used an exploratory factor analysis and proposed 3 subtypes of Cotard's syndrome: psychotic depression, Cotard type I, and Cotard type II. The psychotic depression that patients mostly manifested involved melancholia and some nihilistic delusions. Cotard type I patients are classified as having pure Cotard's syndrome and being grossly delusional rather than having an affective disorder; Cotard type II patients are classified as a mixed group with anxiety, depression, and auditory hallucinations.

 ECT in our patient effectively released her from psychomotor retardation, appetite loss, and Cotard's delusion, which might constitute the psychotic depression subtype of Berrios and Luque [14]. In this sense, the patient had depression with comorbid dementia: the former was treated with ECT, and the latter remained. However, the extent to which the patient's inactivity consisted of depressive inhibition is unclear.

 On the other hand, the effectiveness of ECT for Cotard's delusion seems to have originated from its effect on the depression. If our patient's type of Cotard's syndrome was Cotard type I or II, ECT might be effective for Cotard's delusion, because ECT potentially has an effect on psychotic symptoms. The mechanism of effectiveness of ECT for Cotard's delusion is not known to date.

 Some studies suggest that Cotard's syndrome has neurological correlates. In a review by Kudlur et al. [15], most CT/MRI studies of Cotard's syndrome noted, although not always consistently, abnormalities in the nondominant frontal, temporal, and occasionally the parietal lobes. While these findings may suggest a relation between Cotard's syndrome and FTD, it is unlikely that ECT has an effect on the organic brain dysfunction.

 The effectiveness of ECT on organic brain dysfunction including dementia is an important issue to consider in the case of depression-dementia medius. While its effectiveness for depressive symptoms is in no doubt, evidence of its efficacy for dementia remains uncertain. Rather, ECT may cause adverse cognitive effects and even neuronal damage [16]. However, Palmio et al. measured biomarkers of neuronal injury before and after ECT and concluded that ECT does not cause neuronal injury [17]. Moreover, case reports of patients with depression and dementia or cognitive impairment treated with ECT showed that depression was of course improved, with either no adverse effect on dementia [18] or even improvement [19, 20].

## 4. Conclusion

These findings suggest that ECT is not contraindicated in patients with dementia, and the positive outcome in the present case illustrates that ECT can benefit patients with marked and prolonged depression.
